# Knockdown of SHP-2 delays renal tubular epithelial cell injury in diabetic nephropathy by inhibiting NLRP3 inflammasome-mediated pyroptosis

**DOI:** 10.1515/biol-2025-1190

**Published:** 2025-11-20

**Authors:** Panli Tian, Yanli Ma, Tao Shang

**Affiliations:** Department of Endocrinology, People’s Hospital of Ningxia Hui Autonomous Region, Ningxia Medical University, Yinchuan, Ningxia Hui Autonomous Region, 750002, China; Department of Cardiovascular, People’s Hospital of Ningxia Hui Autonomous Region, Ningxia Medical University, No. 301, Zhengyuan North Street, Jinfeng District, Yinchuan, Ningxia Hui Autonomous Region, 750002, China

**Keywords:** SHP-2, diabetic nephropathy, renal tubular epithelial cell, NLRP3, pyroptosis

## Abstract

Src homology phosphotyrosyl phosphatase 2 (SHP-2) has been implicated in the pathogenesis of diabetic nephropathy (DN), while pyroptosis, an inflammatory form of programmed cell death, has also been associated with disease progression. However, the regulatory interplay between SHP-2 and pyroptosis in DN remains incompletely understood. In this study, we established DN rat models using a single intraperitoneal injection of streptozotocin (STZ) and HK-2 cells cultured under high-glucose (HG) conditions. Hematoxylin and eosin staining was performed to assess the histopathological changes in renal tissues, while immunofluorescence and Western blotting were used to evaluate SHP-2 and NLRP3 expression in both rat kidney tissues and HK-2 cells. Lentiviral transfection was performed to overexpress SHP-2 or NLRP3, following which the expression of pyroptosis-related proteins, activation of the NLRP3 inflammasome, and cell apoptosis were assessed by Western blot and flow cytometry. The results demonstrated that STZ-treated rats exhibited significant weight loss, hyperglycemia, and renal tissue injury. We observed an increase in SHP-2 expression in the kidney tissues of DN rats and in HK-2 cells exposed to high glucose, along with an elevated expression of NLRP3. SHP-2 knockdown suppressed NLRP3 inflammasome activation and mitigated HG-induced pyroptosis in renal tubular epithelial cells. Notably, overexpression of NLRP3 partially reversed the protective effects conferred by SHP-2 knockdown. These findings suggest that SHP-2 knockdown alleviates renal tubular epithelial cell injury in DN by inhibiting NLRP3 inflammasome-mediated pyroptosis.

## Graphic abstract

**Figure:**
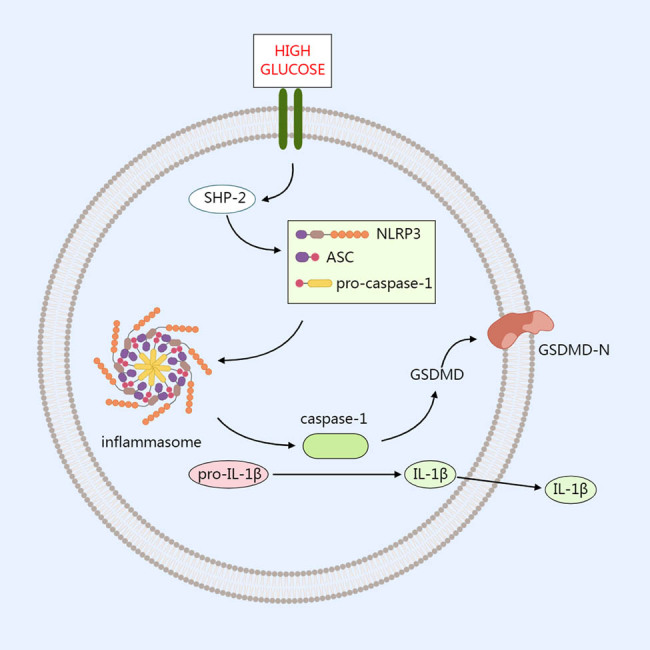

## 1 Introduction

Diabetes mellitus (DM) is a complex metabolic disorder characterized by dysregulated energy metabolism. Its complications, including diabetic neuropathy (DN), cardiovascular disease, severely impair patients’ quality of life and remain difficult to manage clinically [1]. Among these, DN is a leading cause of chronic kidney disease and end-stage renal disease, and significantly contributes to the high disability and mortality rates associated with DM [2]. Thus, understanding the pathogenic mechanisms underlying DN is therefore essential for the development of effective therapeutic strategies. It is well established that immune and inflammatory responses play a central role in the onset and progression of DN, particularly in the context of renal tubular injury [3]. Pyroptosis, a form of programmed cell death driven by inflammatory signaling, has been increasingly recognized in this process. In particular, NLRP3 inflammasome-mediated pyroptosis has been shown to contribute to DN pathogenesis [4].

Src homology phosphotyrosyl phosphatase 2 (SHP-2), encoded by the PTPN11 gene, belongs to the non-receptor tyrosine phosphatase family and is essential for maintaining cellular function [5]. SHP-2 is involved in the regulation of inflammatory responses, cell survival, and metabolic homeostasis, among other processes [6]. In a high-glucose (HG) environment, elevated uric acid may activate the ROS/NLRP3/SHP-2 signaling pathway, thereby promoting epithelial–mesenchymal transition in renal tubular epithelial cells and accelerating the development of renal fibrosis in DN [7]. Similarly, in sepsis-associated encephalopathy, Nogo-A has been shown to exacerbate disease progression by disrupting the SHP-2/NLRP3 balance in microglia through reactive oxygen species (ROS) generation and M1 polarization [8]. In renal carcinoma, allosteric inhibition of SHP-2 has been found to induce caspase-1-dependent pyroptosis to enhance interferon-α-mediated antitumor immunity [9].

Despite these findings, the role and underlying mechanisms of SHP-2 in renal tubular injury in DN remain poorly defined. To address this gap, the present study aimed to investigate the functional role of SHP-2 in tubular epithelial injury in DN using an *in vitro* model, with the goal of elucidating potential molecular pathways involved.

## 2 Methods

### 2.1 Animal experimental design

All animal procedures were approved by the Laboratory Animal Ethics Committee (Changsha, China) and were conducted in accordance with internationally accepted ethical guidelines. Briefly, male C57BL/6 mice aged 8 weeks were randomly assigned to two groups: a streptozotocin (STZ) group and a control group (*n* = 6 per group). Both groups were housed under identical conditions for 12 weeks. To induce DN, mice in the STZ group received a single intraperitoneal injection of STZ at a dose of 60 mg/kg [10]. Blood glucose levels were monitored via tail vein sampling. Mice with blood glucose levels exceeding 16.7 mM for three consecutive days were considered to have successfully developed DN.


**Ethical approval:** The research related to animal use has been complied with all the relevant national regulations and institutional policies for the care and use of animals, and has been approved by the Ethics Committee of People’s Hospital of Ningxia Hui Autonomous Region (Approval no. 2023-NZR-063).

### 2.2 Hematoxylin and eosin (H&E) staining

Kidney tissues were fixed overnight in 4% paraformaldehyde (pH 7.4), embedded in paraffin, and sectioned into 5 μm serial slices. These sections were stained with H&E to assess histopathological changes. Morphological alterations in the glomeruli and tubulointerstitium were observed under a fluorescence microscope (Olympus, Nanjing).

### 2.3 Immunofluorescence

Fixed tissues were permeabilized with PBS containing 0.5% Triton X-100. After blocking with 3% bovine serum albumin for 1 h, the tissues were incubated overnight at 4°C with primary antibodies against SHP-2 (1:100, ab300579, Abcam) and NLRP3 (1:200, ab263899, Abcam). The next day, the tissues were incubated for 1 h at room temperature in the dark with a fluorescently labeled secondary antibody (Alexa Fluor^®^ 488; ab150077, Abcam), the nuclei were counterstained with DAPI for 10 min, and fluorescence signals were visualized using a fluorescence microscope.

### 2.4 Cell culture and treatment

Human renal proximal tubular epithelial cells (HK-2) (ATCC, USA) were cultured in DMEM/F12 medium (Gibco) supplemented with 10% fetal bovine serum and antibiotics, in a humidified incubator at 37°C with 5% CO_2_. When cells reached approximately 80% confluence, they were treated with HG (30 mM) and divided into four groups: (A) Control, (B) HG group, (C) HG + shNC group, and (D) HG + shSHP-2 group.

### 2.5 Transfection experiments

The short hairpin RNA (shRNA) targeting SHP-2 (sequence: 5′-GGGCCAGAGCAGTCAGTAA-3′) was obtained from Shanghai Gene Pharmaceutical Co., Ltd. HK-2 cells were transfected using Lipofectamine^®^ 3000 reagent (Invitrogen, USA), with a scrambled shRNA (shNC) used as the negative control. After transfection, the cells were exposed to 30 mM D-glucose for 24 h, and in a separate experiment, SHP-2–silenced HK-2 cells (shSHP-2) were transfected with the pcDNA3.1-NLRP3 overexpression vector (oe-NLRP3) using the same reagent. Overall, the transfection period for all groups was 48 h.

### 2.6 Flow cytometry

Flow cytometric analysis was performed to evaluate programmed cell death using an Annexin V-FITC/PI apoptosis detection kit (Beyotime). Adherent cells were collected and stained in the dark with 5 µL Annexin V-FITC and 10 µL propidium iodide. The samples were then analyzed using a CytoFLEX flow cytometer (Beckman Coulter, USA), and the results were assessed using the CytExpert 2.1 software (Beckman Coulter, USA).

### 2.7 Western blotting

Total proteins were extracted from HK-2 cells using RIPA lysis buffer (Beyotime). Protein concentrations were determined using a BCA protein assay kit (Beyotime). Equal amounts of protein were separated by SDS-PAGE and transferred onto PVDF membranes (Millipore, MA, USA), the membranes were blocked with 5% skim milk for 2 h at room temperature and then incubated overnight at 4°C with the following primary antibodies: SHP-2 (ab300579), NLRP3 (ab263899), ASC (ab309497), pro-caspase-1 (ab179515), IL-1β (ab283818), GSDMD (ab219800), and GAPDH (ab9485), all from Abcam. After washing, the membranes were incubated with appropriate secondary antibodies (Proteintech, Wuhan, China) for 1 h at room temperature. Immunoreactive bands were visualized using enhanced chemiluminescence reagents (Millipore, MA, USA).

### 2.8 Statistical analysis

Statistical analyses were performed using SPSS version 20.0 (IBM Corp., Armonk, NY, USA). Data are presented as mean value ± standard deviation (SD). Differences among multiple groups were assessed using one-way analysis of variance, and comparisons between two groups were conducted using Student’s *t*-test. A *P*-value <0.05 was considered to indicate statistical significance.

## 3 Results

### 3.1 SHP-2 is highly expressed in the kidney tissue of diabetic mice

**Figure 1:**
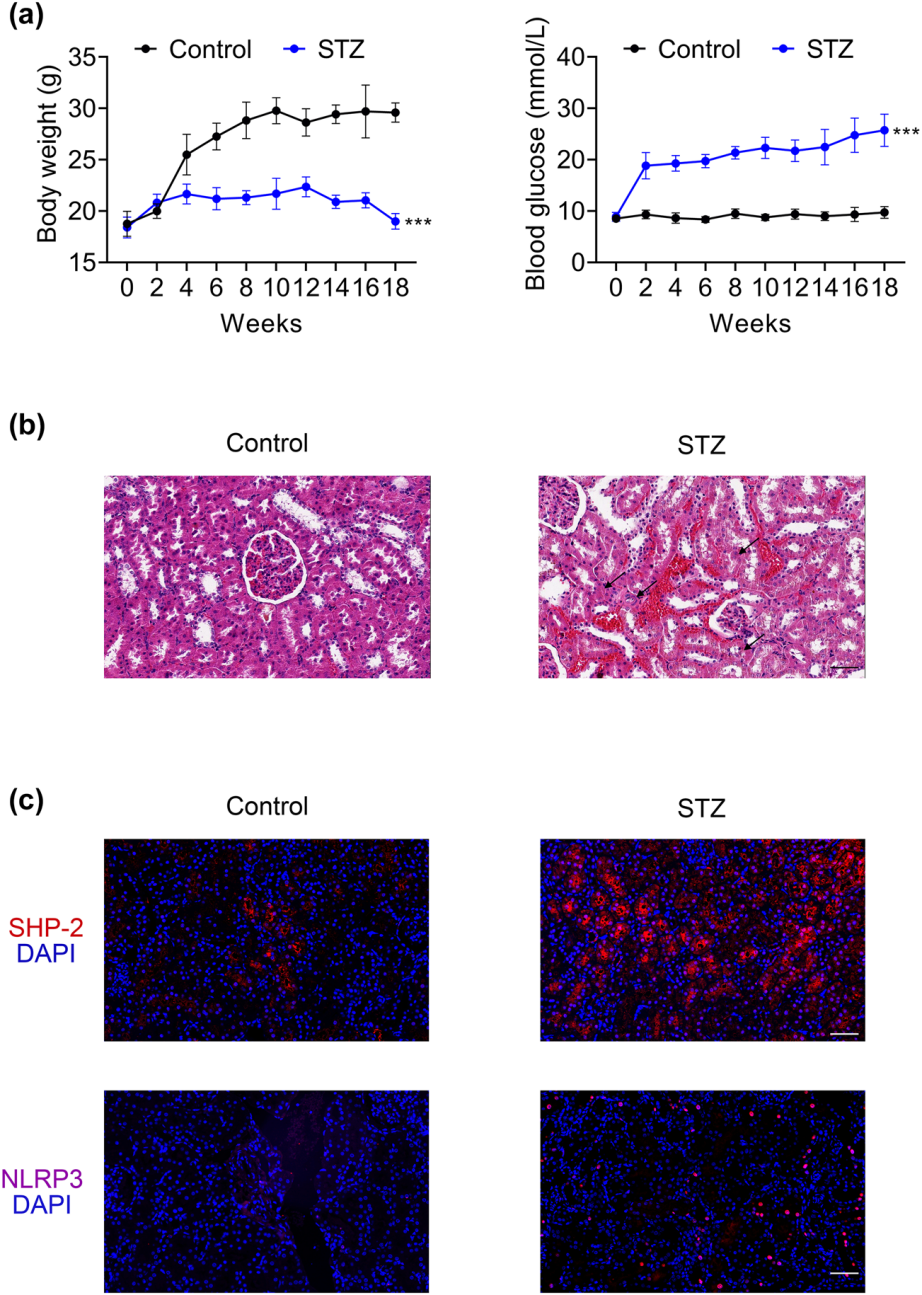
SHP-2 is highly expressed in the kidney tissue of diabetic mice. (a) Body weight and blood glucose levels in mice. (b) H&E staining of kidney tissues to assess pathological changes. (c) Immunofluorescence analysis of SHP-2 and NLRP3 expression in kidney tissue. Values are presented as mean value ± SD. ***p < 0.001 vs Control. *n* = 6.

After STZ injection, the mice exhibited a significant reduction in body weight along with a marked elevation in blood glucose levels, confirming the successful establishment of the diabetic mouse model (Figure 1a). H&E staining further validated the DN model, revealing disappearance of the proximal renal tubular brush border and the presence of vacuolar degeneration in the kidneys of STZ-treated mice compared to the control group (Figure 1b). Immunofluorescence analysis demonstrated increased expression of SHP-2 and NLRP3 in the renal tissues of STZ-treated mice (Figure 1c), indicating that both proteins may be involved in renal tubular injury associated with DN.

### 3.2 SHP-2 expression is elevated in HK2 cells treated with high glucose

**Figure 2:**
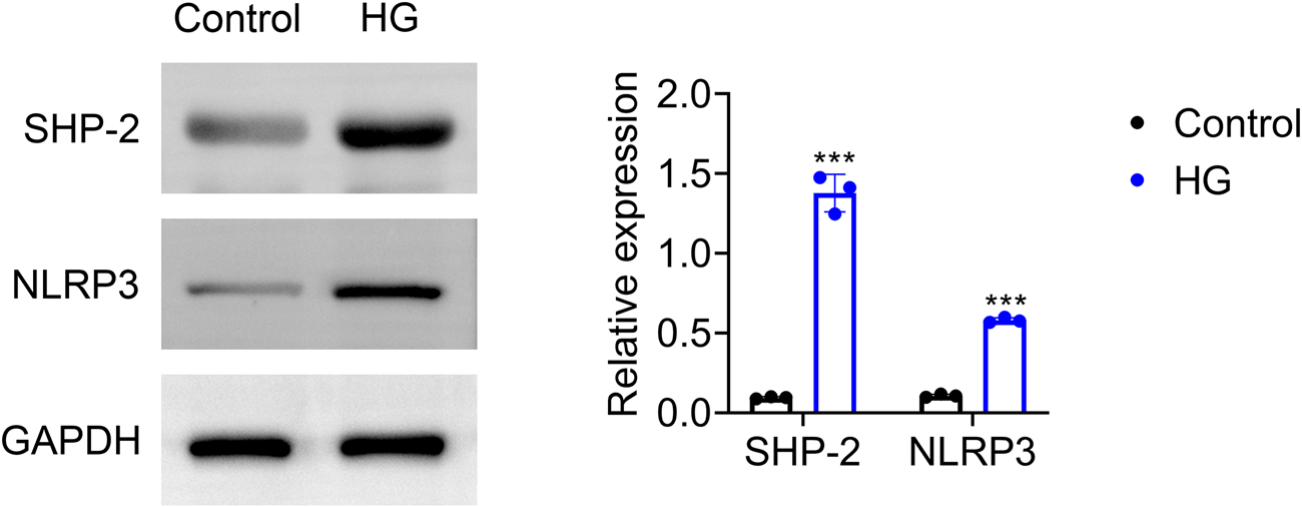
SHP-2 expression is elevated in HK-2 cells treated with high glucose. Expression of SHP-2 and NLRP3 in HK-2 cells following HG treatment. Values are presented as mean value ± SD. ***p < 0.001 vs Control. *n* = 3.

To further investigate the role of SHP-2 in DN, we utilized HG treatment to simulate diabetic conditions in HK-2 cells. Consistent with the *in vivo* findings, HG exposure led to elevated expression of SHP-2 and NLRP3 in HK-2 cells (Figure 2), suggesting a potential involvement of SHP-2 in HG-induced renal tubular epithelial cell injury.

### 3.3 Knockdown of SHP-2 inhibits pyroptosis in HK2 cells treated with high glucose

**Figure 3:**
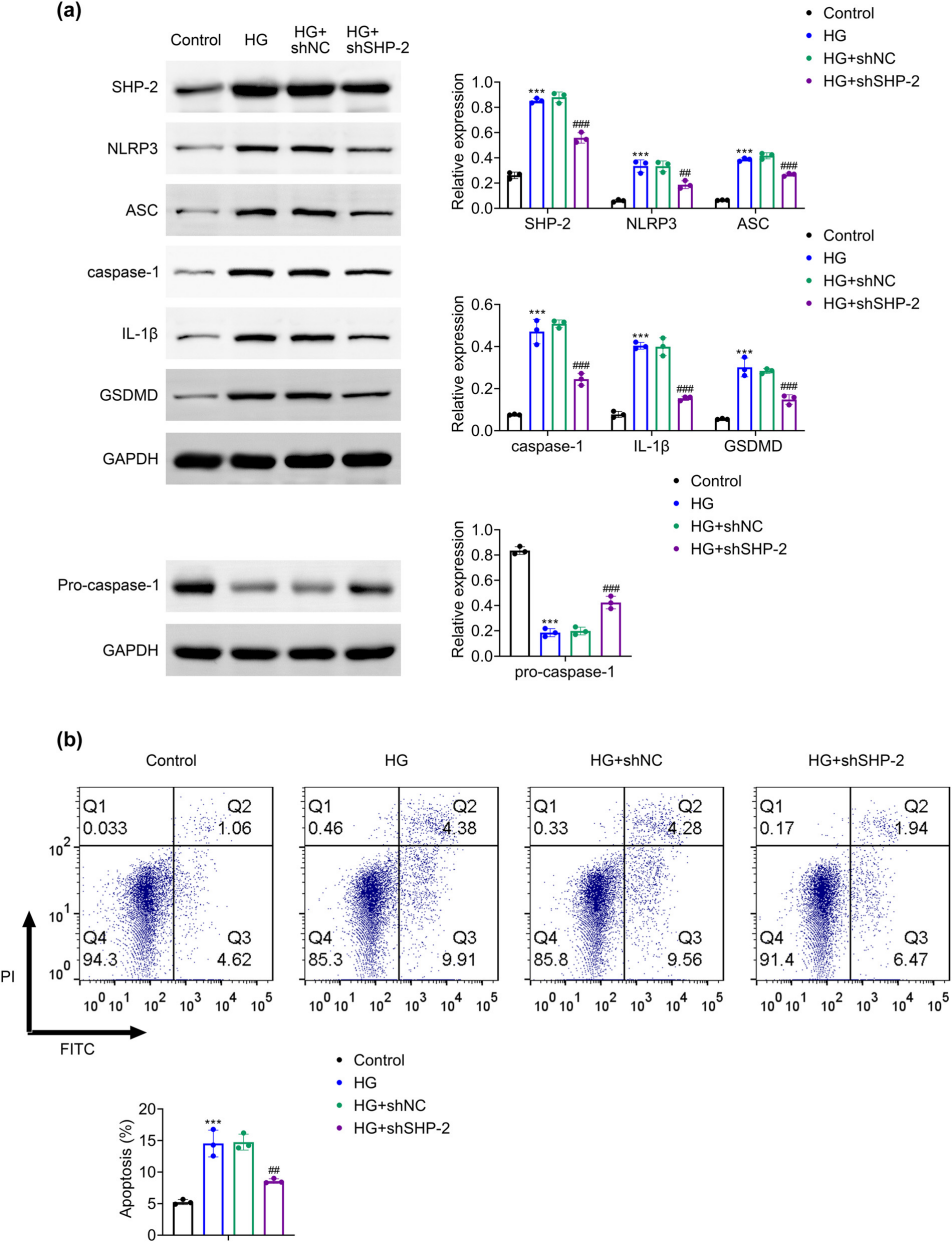
Knockdown of SHP-2 inhibits pyroptosis in HK-2 cells treated with high glucose. (a) Western blot analysis of SHP-2, NLRP3, ASC, pro-caspase-1, caspase-1, IL-1β, and GSDMD expression. (b) Flow cytometric analysis of apoptosis rate. Values are presented as mean value ± SD. ***p < 0.001 vs Control. ^##^ *p* < 0.01, ^###^ *p* < 0.001 vs HG + shNC. *n* = 3.

Given the known regulatory role of SHP-2 in pyroptosis under various physiological and pathological conditions, we examined its function in HG-induced pyroptosis in HK-2 cells. The results indicated that SHP-2 knockdown effectively reduced SHP-2 expression under HG conditions (Figure 3a). Notably, the HG-induced upregulation of caspase-1, GSDMD, IL-1β, and inflammasome components, including NLRP3, ASC, and pro-caspase-1, was significantly attenuated after SHP-2 silencing (Figure 3a). In addition, SHP-2 knockdown significantly reduced HG-induced apoptosis in HK-2 cells (Figure 3b). These results indicate that SHP-2 promotes HG-induced pyroptosis and apoptosis in renal tubular epithelial cells.

### 3.4 Activation of NLRP3 reverses the effects of overexpressed SHP-2

**Figure 4:**
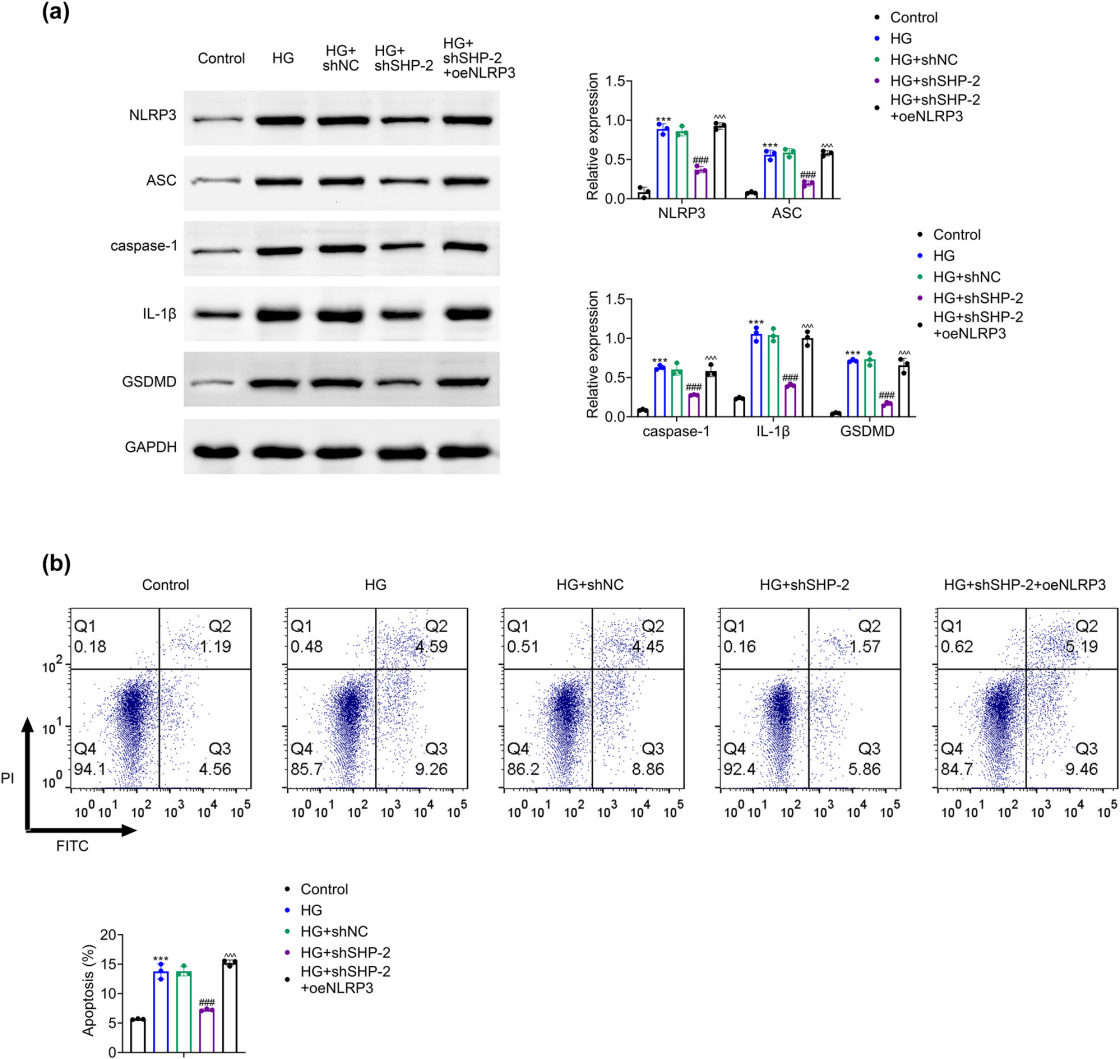
Activation of NLRP3 reverses the effects of SHP-2 knockdown. (a) Western blot analysis of NLRP3, ASC, caspase-1, IL-1β, and GSDMD expression. (b) Flow cytometric analysis of apoptosis rate. Values are presented as mean value ± SD. ***p < 0.001 vs Control. ^###^ *p* < 0.001 vs HG + shNC. ^^^^^ *p* < 0.001 vs HG + shSHP-2. *n* = 3.

To determine whether NLRP3 mediates the pro-pyroptotic effects of SHP-2, we overexpressed NLRP3 in SHP-2-silenced HK-2 cells. Western blot analysis confirmed successful overexpression of NLRP3. Compared with SHP-2 knockdown alone, NLRP3 overexpression restored the expression of pro-caspase-1, IL-1β, ASC, and GSDMD and partially inhibited the anti-apoptotic effects observed in SHP-2–silenced cells (Figure 4). Collectively, these findings suggest that knockdown of SHP-2 suppresses the activation of the NLRP3 inflammasome, thereby attenuating HG-induced pyroptosis in renal tubular epithelial cells, while NLRP3 overexpression can partially reverse this protective effect.

## 4 Discussion

DN is one of the leading causes of chronic renal failure, and its pathogenesis involves multiple contributing factors. Despite ongoing research, the underlying pathophysiological mechanisms of DN remain incompletely elucidated [11]. Pyroptosis, a recently recognized form of programmed cell death distinct from necrosis and apoptosis, has been implicated in the development and progression of diabetes and its complications, including DN [4,12].

Given the emerging role of SHP-2 in renal pathophysiology, we investigated its involvement in pyroptosis in the context of DN, as the regulatory mechanisms underlying pyroptosis in this setting are not yet fully defined. Previous studies have demonstrated that SHP-2 is upregulated in diabetic kidneys and contributes to renal inflammation in diabetic rats [13]. Moreover, macrophage-specific SHP-2 deficiency has been shown to attenuate DN by suppressing inflammation mediated through the MAPK and NF-κB pathways [14], further supporting the association between SHP-2 and DN pathogenesis. Additionally, inhibition of the SHP-2/PI3K/NLRP3 axis has been reported to reduce pyroptosis and ameliorate non-alcoholic steatohepatitis [15], while SHP-2 has also been implicated in promoting pyroptosis and activating the NLRP3 inflammasome in renal cell carcinoma [9]. However, the relationship between SHP-2 and pyroptosis in renal tubular epithelial cells under diabetic conditions had not been previously established. In this present study, we found that SHP-2 expression was upregulated in DN mice kidney tissues and HG-treated HK-2 cells, consistent with earlier reports. Importantly, SHP-2 knockdown significantly reduced HG-induced pyroptosis in renal tubular epithelial cells, suggesting that SHP-2 overexpression may serve as a key initiator of pyroptosis in DN.

Pyroptosis is a distinct form of inflammatory cell death mediated by caspase-1 and regulated via the GSDMD signaling cascade [16]. It is typically initiated by inflammasome activation, with the NLRP3 inflammasome playing a central role in the inflammatory response and pyroptotic cell death [17]. GSDMD is the primary executioner of pyroptosis; upon inflammasome activation, it is cleaved by caspase-1 or caspase-11 to release the N-terminal fragment (GSDMD-N), and this active fragment binds to phospholipids in the cell membrane, forming pores that lead to cell swelling and lysis, leading to the disruption of membrane integrity, the release of inflammatory mediators, and cell death in response to microbial invasion or endogenous danger signals [18].

Although our studies demonstrate that SHP-2 knockdown alleviates pyroptosis by inhibiting the NLRP3 inflammasome, the upstream mechanisms regulating SHP-2 expression in DN remain to be fully elucidated. Previous studies have shown that SHP-2 is regulated by multiple factors under diabetic conditions. For example, previous studies have shown that SHP-2 is regulated by multiple factors under diabetic conditions. For example, ROS have been shown to activate SHP-2 through oxidative modification or activation of upstream kinases [19]. Furthermore, in the local inflammatory microenvironment of DN, inflammatory cell infiltration leads to increased profibrotic cytokine pressure, which may partly explain the activation of SHP-2 in DN [13]. Future studies aiming to identify the precise upstream regulators of SHP-2 in renal tubular cells will further enhance our understanding of its role in the pathogenesis of DN and may provide new therapeutic targets.

Our findings further demonstrated that NLRP3 overexpression partially reversed the inhibitory effects of SHP-2 knockdown on pyroptosis in HG-treated HK-2 cells, indicating that SHP-2 induces pyroptosis of renal tubular epithelial cells through NLRP3.

## 5 Conclusion

In summary, our findings indicate that SHP-2 expression is increased in both DN mice and HK-2 cells treated with HG, and its knockdown reduced HG-induced pyroptosis in HK-2 cells, an effect that appeared to be partly mediated by the NLRP3 pathway, which could then prevent the progression of DN.
